# Pilot Randomized Trial of the Effect of Wireless Telemonitoring on Compliance and Treatment Efficacy in Obstructive Sleep Apnea

**DOI:** 10.2196/jmir.9.2.e14

**Published:** 2007-05-17

**Authors:** Carl J Stepnowsky, Joe J Palau, Matthew R Marler, Allen L Gifford

**Affiliations:** ^3^California Institute for Telecommunications and Information Technology (Calit2)University of CaliforniaIrvineCAUSA; ^4^VA Bedford Center for Health Quality, Outcomes & Economic ResearchVA New England HealthcareBedfordMAUSA; ^5^Department of Public Health and MedicineBoston UniversityBostonMAUSA; ^2^Department of PsychiatryUniversity of CaliforniaSan DiegoCAUSA; ^1^Health Services Research & Development ServiceVeterans Affairs San Diego Healthcare System (VASDHS)San DiegoCAUSA

**Keywords:** Continuous positive airway pressure therapy, CPAP, sleep apnea syndromes, treatment compliance, telemedicine, randomized controlled trial

## Abstract

**Background:**

Obstructive sleep apnea (OSA) is a prevalent and serious medical condition characterized by repeated complete or partial obstructions of the upper airway during sleep and is prevalent in 2% to 4% of working middle-aged adults. Nasal continuous positive airway pressure (CPAP) is the gold-standard treatment for OSA. Because compliance rates with CPAP therapy are disappointingly low, effective interventions are needed to improve CPAP compliance among patients diagnosed with OSA.

**Objective:**

The aim was to determine whether wireless telemonitoring of CPAP compliance and efficacy data, compared to usual clinical care, results in higher CPAP compliance and improved OSA outcomes.

**Methods:**

45 patients newly diagnosed with OSA were randomized to either telemonitored clinical care or usual clinical care and were followed for their first 2 months of treatment with CPAP therapy. CPAP therapists were not blinded to the participants’ treatment group.

**Results:**

20 participants in each group received the designated intervention. Patients randomized to telemonitored clinical care used CPAP an average of 4.1 ± 1.8 hours per night, while the usual clinical care patients averaged 2.8 ± 2.2 hours per night (*P* = .07). Telemonitored patients used CPAP on 78% ± 22% of the possible nights, while usual care patients used CPAP on 60% ± 32% of the nights (*P* = .07). No statistically significant differences between the groups were found on measures of CPAP efficacy, including measures of mask leak and the Apnea-Hypopnea Index. Patients in the telemonitored group rated their likelihood to continue using CPAP significantly higher than the patients in the usual care group. Patients in both groups were highly satisfied with the care they received and rated themselves as “not concerned” that their CPAP data were being wirelessly monitored.

**Conclusions:**

Telemonitoring of CPAP compliance and efficacy data and rapid use of those data by the clinical sleep team to guide the collaborative (ie, patient and provider) management of CPAP treatment is as effective as usual care in improving compliance rates and outcomes in new CPAP users. This study was designed as a pilot—larger, well-powered studies are necessary to fully evaluate the clinical and economic efficacy of telemonitoring for this population.

## Introduction

Sleep-disordered breathing is a generic diagnostic term broadly used to describe apnea (cessation of airflow), hypopnea (reduction in airflow), and other breathing irregularities that occur during sleep. It is a common, albeit underdiagnosed, chronic condition in the adult population, with up to 4% of females and 9% of males experiencing at least 15 episodes of apnea and/or hypopnea per hour of sleep [1-4].The sleep-disordered breathing syndrome known as obstructive sleep apnea/hypopnea (OSA) entails repetitive occlusion or narrowing of the upper airway, which results in intermittent decreases in oxyhemoglobin (blood oxygen desaturation) and increases in blood carbon dioxide levels and, in turn, leads to frequent arousals from sleep. The primary and most measured symptom of OSA is the daytime drowsiness and fatigue that follow the nightly sleep disruption. The seriousness of OSA, however, is underscored in aggregate clinical profile by significant comorbidities, including obesity, depression, ischemic heart disease, stroke, hypertension, and increased risk of automobile-related accidents, and by the extreme outcome of premature death [1,5,6].

Nasal application of continuous positive airway pressure (CPAP) [7] is the treatment of choice for this condition, involving use of both an air flow generator and a mask to supply a constant stream of pressure through the pharyngeal airway during sleep [8]. Despite the documented clinical efficacy of CPAP, many—perhaps most—patients have difficulty adhering to treatment owing to one or more of a variety of discomforting side effects such as pressure intolerance, claustrophobic reaction to the nasal mask, mask dislodgement, and machine noise. Estimates of the noncompliance rates among OSA patients who start a CPAP regimen but terminate treatment within one year are as high as 50% or more [9]. Among patients who persevere to the one year mark or later, most demonstrate only partial compliance by not using CPAP for the entire night as prescribed. Published objective compliance rates from studies conducted in the United States range from 3.3 to 5.3 hours per night [10-12], with one study showing that only 6% of patients used the machine for ≥ 7 hours per night on at least 70% of the nights monitored [10]. As these study findings clearly indicate, effective interventions are needed to improve CPAP compliance among the OSA-diagnosed patient population at large.

Over the past decade, a variety of psychoeducational and technological interventions designed to improve CPAP compliance have been developed and tested. To date, the scope of psychoeducational interventions includes telephone follow-ups with dissemination of OSA-CPAP literature to patients [13], intensive patient education and support protocols [14-16], and motivational enhancement programs [17]. While most of these approaches have had only modest impact on compliance, the intensive support protocols have had greater impact but require a substantial amount of time and resources to implement. Technological advances include more comfortable interfaces, variable positive airway pressure (PAP) levels responsive to the respiratory cycle, and humidification. Although clinical logic and experience suggest that these technological improvements can enhance compliance by increasing patient comfort, a large-scale systematic review of the applications and outcomes reveals that they, on average, result in little, if any, effect on CPAP compliance rates [18]. In the present study, the aim was to achieve better CPAP compliance and clinical efficacy by combining and integrating the most promising elements of both psychoeducational interventions and technological innovations. This design imperative led us to deploy a recently developed telemedicine system that allows tailored management of OSA-CPAP patients through wireless monitoring of “time at prescribed pressure” and transmission of those CPAP compliance- and efficacy-relevant data to care providers in 24-hour cycles.

This pilot intervention was informed and shaped by three major trends in the findings of prior studies, each predicated on a departure from usual care for OSA-CPAP patients. Usual care entails initial patient-specific titration of the CPAP device to the critical pressure needed to keep the upper airway open during sleep, a follow-up telephone call from the CPAP provider or sleep physician’s office to check on the patient’s comfort and usage within one week of CPAP initiation in the home environment, and subsequent in-office visits starting several weeks after CPAP initiation and continuing thereafter as needed. The first salient trend in prior findings is that patients’ self-reported difficulties with CPAP as well as their subjective compliance do not provide sufficient or reliable information to guide appropriate clinical management of OSA under usual care conditions. For example, engaged in a novel therapy, a patient may not be aware of excessive mask leakage during the initial week of CPAP and so will fail to report the problem during the one-week follow-up call. Uncorrected mask leakage, by hampering critical pressure delivery, can have a seriously deleterious impact on compliance. The second trend is that objective compliance and efficacy data are typically not obtained in a timely manner. There are, in general, three standard patient-dependent modes by which a clinical sleep team obtains the objective compliance data recorded as machine-on time by the CPAP unit: (1) the patient brings the CPAP unit into the office and the sleep team physically downloads the data, (2) the patient transmits the data from home via a telephone line, and (3) the patient removes a memory card from the CPAP unit and either mails it in or brings it into the office during a follow-up visit. Much as compliance with medical regimens can be problematic [19], patient transfer of the CPAP data to the clinical team can be equally erratic. The third trend is that compliance patterns are established early in treatment initialization, so time and problem resolution are of the essence in the effort to establish a pattern of treatment compliance [20]. A sleep team’s ability to detect problems and intervene early in therapy may improve a patient’s early response to CPAP therapy and increase the likelihood that the patient will become a regular and compliant user, thereby enhancing clinical outcome.

Given the very limited number of prior CPAP compliance- and efficacy-related studies within the telemedicine arena, the present study breaks new ground by attempting to counter the known shortcomings of usual care in both telemonitoring design and to answer the overarching question: Does more quickly delivered advice and counsel from providers to patients about developing objectively measured in-home compliance patterns and problems translate to enhanced treatment compliance levels sufficient for improvements in patient health status and outcomes?

## Methods

### Design

This was a randomized controlled pilot study approved by the local Institutional Review Board. Newly diagnosed OSA patients who met inclusion criteria were asked to participate. Patients were randomized to either the usual clinical care (UCC) group or the telemonitored clinical care (TCC) group. Both groups of patients received the monitoring device and were followed for an intervention period of 2 months.

### Participants

Participants were patients at the Veterans Affairs San Diego Healthcare System (VASDHS) who were referred to the sleep clinic by their physician for suspicion of OSA. All patients had their sleep recorded with the Stardust sleep recording system (Respironics, Pittsburgh, PA), which monitors heart rate, oximetry, nasal airflow, chest wall movement, and body position. Patients diagnosed with OSA based on the results of their sleep study and clinical history were recruited, consented, and enrolled if they met all of the following criteria: diagnosis of moderate-to-severe OSA, defined as an Apnea-Hypopnea Index (AHI) ≥ 15 events per hour; naive to CPAP therapy; stable sleep environment (operationally defined as a permanent address, requisite for wireless monitoring); and at least 18 years of age. An AHI of ≥15 was chosen in an effort to be consistent with current OSA guidelines and practice parameters [8,9,21,22].

Patients were excluded from the study if they met any one of the following criteria: allergies or sensitivity to the mask or mask material; previous use of any other PAP device (eg, bi-level PAP, auto-adjusting PAP); current use of prescribed supplemental oxygen; or significant comorbid medical conditions that would prevent the patient from completing the protocol. Significant comorbidities were defined as any medical or mental health condition that could interfere with the daily use of CPAP. Additionally, patients were excluded if they lived in a geographically unsuitable region (ie, outside of the wireless network coverage area). A total of 91 patients at the VASDHS Sleep Clinic either signed or gave verbal consent to be contacted so they could learn more about the study. From these 91 patients, 46 were either were not interested in study participation or did not satisfy the inclusion and exclusion criteria.

**Figure 1:**
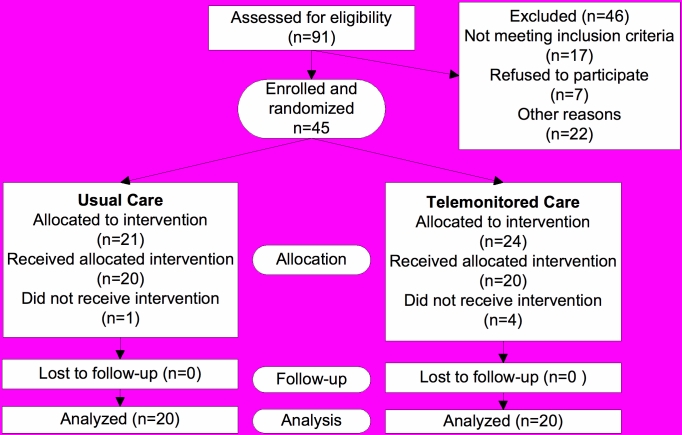
Participant flow and randomization chart

The remaining 45 patients signed consent forms and were enrolled into the study. The study took place from October 2004 to August 2006. Figure 1 illustrates the patient flow and randomization scheme. The randomization scheme was concealed until the time at which the intervention was assigned. The randomization scheme was generated by the project statistician and carried out by research staff immediately after the informed consent procedure and the completion of the baseline questionnaires. Using random assignment, 21 patients were assigned to the TCC arm and 24 patients to the UCC arm, with 20 participants in each group receiving the designated intervention. There were five CPAP “rejectors,” or patients who decided within the first day or two that they did not want to pursue CPAP as the primary treatment for their OSA. Our study did not have a “run-in” period, which could have helped identify these patients prior to the intervention.

### Apparatus

Each participant was provided with an AutoSet Spirit flow generator unit (ResMed Corp, Poway, CA) set to fixed-mode pressure, which was equipped with the HumidAire 2i heated humidifier (ResMed Corp, Poway, CA). Each participant was provided a compatible nasal or full-face mask; nasal pillows were not used in this study. 

**Figure 2:**
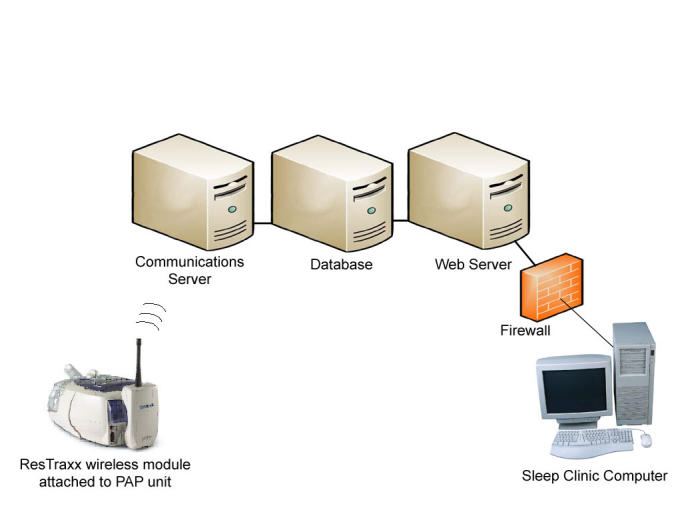
Model of wireless telemonitoring of CPAP compliance and efficacy.

All CPAP flow generators used in the study were outfitted with a ResTraxx wireless transmitter (ResMed Corp, Poway, CA) (Figure 2) to allow the remote data collection of compliance and efficacy information for both groups and to reduce any bias that might be associated with one group (TCC) and not the other (UCC) having the extra attachment. While the patient sleeps, data are collected. The next day, the de-identified data are transmitted to a computer server that meets Health Insurance Portability and Accountability Act (HIPAA) security and privacy standards. The data then become available on a sleep clinic’s computer via connection to a password-protected website where clinicians can access the nighttime CPAP data the following afternoon.

**Figure 3:**
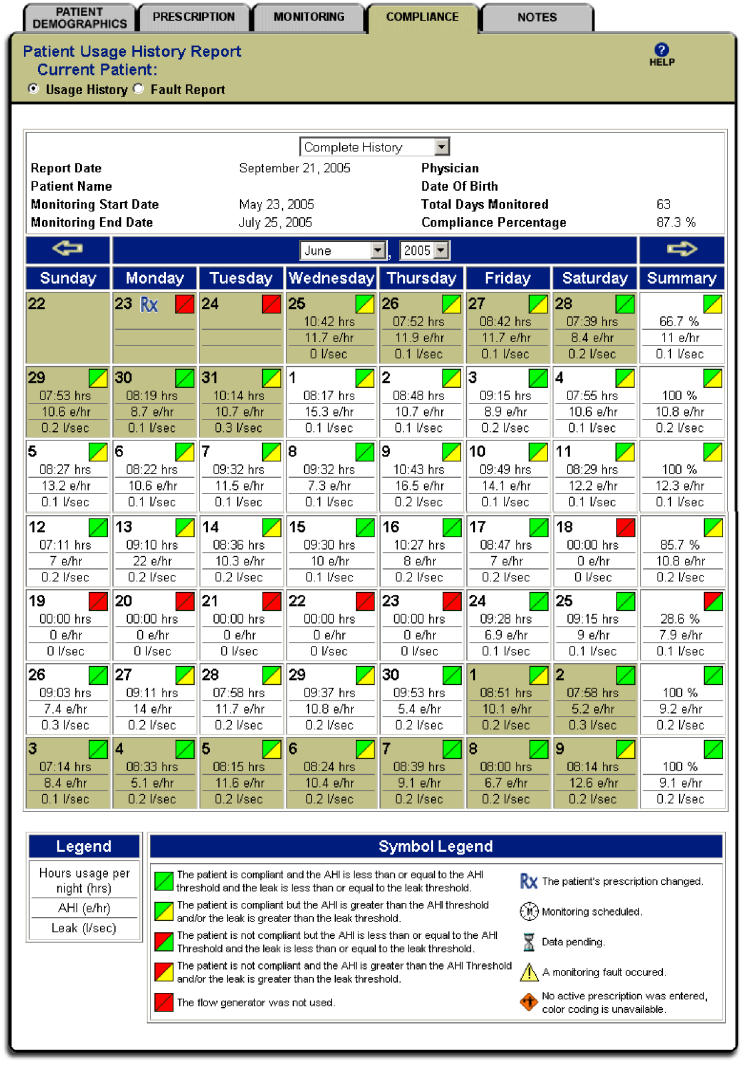
Screenshot of the ResTraxx Data Center website, showing the compliance tab with sample compliance and efficacy data for one month of CPAP use for a hypothetical patient. The thresholds specified on another tab allow for color-coding of efficacy and compliance data — this color-coding allows for quick review of how well any one patient is doing on CPAP. Specific data values are also provided, which can aid clinical management. The symbol legend at the bottom of the tab explains the meaning of each symbol used in this tab (see also Table 2).

Only research and clinical staff had secured access to the data via a standard browser and entry into the ResTraxx Data Center (RDC), the patient and data management website designed for 24/7 access to telemonitored compliance and efficacy data. Per each 24-hour cycle of data transmission, the ResTraxx Data Center website displays the data in a calendar format that reveals daily and weekly data trends. The Multimedia Appendix provides four screenshots of the ResTraxx Data Center website, including patient demographics, prescription, monitoring, and compliance (the latter screen is also shown in Figure 3). Other wireless monitoring systems may be available that allow a similar “passive” procurement of treatment adherence and efficacy data.

### Groups

#### All Participants

Regardless of group assignment, all patients who participated in this study had identical CPAP setups. The initial set-up visit consisted of sleep apnea education, sleep study review, and CPAP instruction and mask-fitting. During this visit, a study staff member educated the patient about sleep apnea and explained the patient’s baseline sleep study results. Each patient was then instructed on how to use CPAP and was fitted for a mask. The patient wore the mask while the CPAP unit was set at the prescribed pressure for approximately 10 minutes for mask adjustment and assessment of pressure tolerance. Both groups were shown how to attach the ResTraxx wireless device to their CPAP units and were provided with written instructions on CPAP use.

#### Usual Clinical Care (UCC) Group

Patients randomized to UCC were treated according to the prevailing standard of care for OSA patients at the VASDHS CPAP Clinic. Usual care consisted of a 1-week telephone call after CPAP initiation and a 1-month in-office follow-up visit by CPAP clinic staff. Patients were encouraged to call the clinic any time they had a problem or concern. CPAP compliance and efficacy data were downloaded at the 1-month time point to help direct clinical management.

#### Telemonitored Clinical Care (TCC) Group

The essence of the TCC intervention is the ability to telemonitor compliance and efficacy data for each patient on a daily basis from the first day of treatment and to act on those data collaboratively, and in partnership, with the patient. Collaborative management refers to the joint decision making and partnership between provider and patient and is characterized by communication, negotiation, and consideration of important patient factors and preferences. Patients in this group had their objective flow generator data monitored as frequently as needed per specified clinical pathways throughout the active 2-month treatment period. The frequency and nature of the clinical interactions depended on both the objectively measured nightly data values and subjective patient reports.

### TCC Parameters and Thresholds

Telemonitoring included review of both compliance and efficacy data. Compliance data encompassed details on how many total hours the PAP unit was used each night at therapeutic pressure. Efficacy data consisted of the amount of mask leakeage (L/s) and the AHI (total number of apneas and hypopneas per hour of sleep). Thresholds for each parameter were set by the study team using the password-secured interactive ResTraxx Data Center website (Table 1).

**Table 1:** Specifications of thresholds for each parameter

Parameter	Threshold
CPAP compliance	4 hours/night
Apnea-Hypopnea Index	10 events/hour of sleep
Mask leak	0.4 L/s

### Website Color-Coding Scheme

The ResTraxx Data Center calendar display (see also Figure 3) summarized the compliance (upper-left diagonal) and AHI / mask leak measures (lower-right diagonal) for each night in a color-coded box-shaped icon. Table 2 presents the color-code scheme of these icons and provides a data description, an interpretation in relation to the threshold value for each parameter (see Table 1), and a recommended general course of action per each actual measure. While each night’s measures were color-coded to ease visual identification of out-of-range values when tracking large numbers of patients, the display for each day contained the specific data values for compliance and efficacy as well. In addition, each week’s display included a summary box providing mean values for each of the parameters for that week.

**Table 2:** Color-coding scheme summarizing compliance and efficacy measures on the ResTraxx Data Center website

Code	Description			Interpretation	Action
green/... 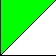	Compliance ≥ 4 hours/night			Compliance at or above threshold	Compliance within limits, no intervention necessary
red/... 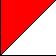	Compliance < 4 hours/night			Compliance below threshold	Consider intervening to increase compliance
.../green 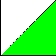	AHI < 10	and	Leak < 0.4 L/s	Leak and AHI below threshold	Efficacy within limits, no intervention necessary
.../yellow 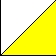	AHI < 10	and	Leak ≥ 0.4 L/s	Either leak or AHI above threshold	Identify which is high and intervene as necessary
		**or**			
	AHI ≥ 10	and	Leak < 0.4 L/s		

Note: The only color code not shown is red/red, which indicates that CPAP was not used on the particular night of monitoring.

### Clinical Pathways

For the purposes of this study, we defined clinical pathways for the interventionists to follow. The pathways specified how frequently the clinical team would check the CPAP compliance and efficacy data values on the ResTraxx Data Center website for each patient. Human monitoring is designed to be more intensive in the earlier stages of treatment, with a gradual tapering off over time if patterns of CPAP compliance are established. A green/green pathway (ie, when all three parameters of compliance, AHI, and mask leak are within normal limits) specified this gradually attenuated monitoring schedule. A red/yellow pathway specified the general course of action required when one or more parameters were not within the normal range of values. In each case, the clinician and the patient collaboratively assessed the source of the problem, considered alternative solutions, and selected a corrective measure. The clinical team then continued close monitoring until each parameter was back within normal range. The red/yellow pathway referred the clinician to an intervention matrix of corrective actions to consider for each problem (Table 3).

**Table 3:** CPAP clinician intervention management matrix for TCC group (adapted from [23])

	Symptoms	Cause	Correction
**Mask**	Dry eyes	Leak	Verify mask fit/size
	Irritated skin	Head gear too tight	Readjust head gear
	Pressure sores	Incorrect mask fit	

**Nasal**	Dry nose, mouth, throat	Lack of humidified air	Use heated humidifier
	Nasal congestion	Mouth breathing	Try chin strap

**Pressure**	Feeling of need for more or less air	Incorrect pressure (too low or high)	Verify prescribed pressure
	Chest discomfort	Claustrophobia	Add short periods of use during the day

**Miscellaneous**	CPAP noise	Blocked air intake	Check air filter
	Bed partner disturbance	Multiple factors	Move unit further from bed
	Still snoring and/or sleepy	Pressure too low	Consider pressure increase

### Data Collection

Questionnaire data were collected at baseline and postintervention. The Measures section below describes each assessment tool and provides operational definitions. Flow generator data from all patients were both wirelessly transmitted to the secure ResTraxx Data Center server and manually downloaded by research staff at the end of the 2-month monitoring period. Data obtained wirelessly were compared to data obtained via manual download to verify accuracy in transmission and confirm the 100% accuracy rate reported previously in the literature for wireless transmission of CPAP data [24]. The study period was composed of 2270 nights for wireless data transmission. However, timely transmission failures occurred on 131 of those nights, 5.7% of the total, owing mainly to minor network issues related to residence location with intermittent coverage. Over the 2088 nights of data collection available for comparison, there were no discrepancies between the two data sources, that is, 100% accuracy was achieved in this study between data obtained wirelessly and data downloaded manually.

### Measures

This study assessed a number of measures both at baseline and postintervention from a number of domains, including sleep study data, CPAP-related data, OSA symptoms, health-related quality of life, and psychological factors (ie, depressive symptoms). Sleep study data were obtained from the baseline Stardust sleep recording system measures that established the diagnosis of OSA and included the AHI. Sleep apnea symptom scales were based on previously published scales of sleep apnea symptom frequency that are reliable, valid, and highly predictive of OSA [25,26]. The Epworth Sleepiness Scale (ESS) [27,28] is a widely used subjective measure of excessive daytime sleepiness that queries likelihood of falling asleep in eight different situations on a scale from 0 (not likely) to 3 (highly likely). Scores on the ESS range from 0 to 24, with higher scores reflective of greater daytime sleepiness. In addition, the research team used a visual analog scale to measure sleepiness. The Center for Epidemiological Studies Depression Scale (CES-D) was used to assess depressive symptomatology [29,30].

A fundamental methodological advantage in studying CPAP compliance is that compliance is measured objectively via a device-internal clock counter. The primary measure used in this study was the number of hours per night that the unit was used at the prescribed pressure. For CPAP device efficacy, three facets of PAP efficacy were measured by the AutoSet Spirit flow generator: mask leak (L/s), pressure (cm H_2_0), and AHI. AHI measurements by AutoSet Spirit have been shown to be highly correlated with the measures recorded by polysomnography [31]. Identical devices were provided to both the TCC and UCC groups to eliminate any potential variation attributable to the use of different devices.

OSA-specific health-related quality of life was assessed using the Functional Outcomes of Sleep Questionnaire (FOSQ). This is a 32-item self-report measure that assesses the impact of disorders of excessive sleepiness on multiple activities of daily living [32].

Clinician satisfaction and patient satisfaction were assessed by questionnaires that include both Likert-type items and open-ended questions. Participants were asked to rate their overall satisfaction with care (1 = poor; 5 = excellent), their likelihood of continuing to use CPAP (1 = not likely; 5 = highly likely), and their concern about being monitored wirelessly (1 = not concerned; 5 = highly concerned).

CPAP self-efficacy refers to OSA patients’ confidence or belief that they can adhere to the regimen necessary to manage their OSA with CPAP. The CPAP self-efficacy scale is a 5-item self-report scale that was developed and validated by the primary author [12].

Communication with the health care team was assessed by a 3-item self-report measure that measures the frequency with which patients use certain communication behaviors, including preparing a list of questions, asking questions about things they want to know and things they don’t understand about their treatment, and discussing any personal problems that may be related to their illness [33]. Respondents are asked to answer using a 6-point Likert-type response ranging from never (0) to always (5).

### Statistical Analysis

For comparison between usual and telemonitored groups at postintervention, an unpaired *t* test was used for normally distributed variables, and the Mann-Whitney test was used for non-normally distributed variables. The improvement of variables from baseline was tested by paired *t* test.

### Power Analysis

We based the power analysis on the effect size from one study that compared intensive clinical support with standard clinical support [15]. The CPAP compliance level in the intensive support group (5.4 ± 1.9 hours/night) was higher than in the standard care group (3.9 ± 2.5 hours/night) over the first month of treatment. In terms of effect sizes, this approximates a d-index of .65. Based on this effect size, an alpha level of .05, and a single-sided hypothesis test, 42 patients would be required to have a power level of .70, which would be an acceptable power level for this pilot study.

The one variable that is difficult to accurately estimate prospectively is the standard deviation (SD) for the treatment compliance; to the extent that SD is high relative to the mean difference, power is lower.

## Results

The participants who completed this study were primarily male (98%), older, and overweight and had moderate-to-severe OSA. Table 4 provides details on the baseline characteristics of the sample and shows that the two groups did not differ on any of the measured scales at baseline. The sample was primarily middle-aged and overweight. In addition, approximately 61% were Caucasian, 15% Black, 10% Filipino, 7% Hispanic, 2% Asian or Pacific Islander, and 5% (self-described as) “other.” All of the participants reported education levels at or above the high school graduate level, with 61% having the equivalent of 14 years of education; 50% of the sample was married; 42% was retired, 37% was working (full or part time), and the remaining 21% was either unemployed, unable to work, or students. The mean (SD) for the education variable was 14.7 years (3.2) and 14.3 years (2.7) for the UCC and TCC groups, respectively. The difference between these means was not statistically different (*P* = .62).

**Table 4:** Baseline patient characteristics and treatment period, by study arm

	Total Group		TCC		UCC		
Characteristic	Mean ± SD	Range	Mean ± SD	Range	Mean ± SD	Range	*P* value^*^
Age (years)	59 ± 14.3	23-80	60 ± 10.8	42-80	58 ± 13.7	23-74	.50
Body mass index (kg/m^2^)	32.8 ± 5.7	26-45	33.3 ± 4.9	25-46	30.5 ± 5.1	25-42	.22
Apnea-Hypopnea Index	39 ± 16.8	21-94.7	44.8 ± 17.9	23.3-93.7	37.6 ± 14.3	21-62.5	.29
CPAP pressure (cm H_2_0)	10.3 ± 1.6	8-13	10.4 ± 1.3	8-13	9.7 ± 1.4	8-13	.29
Sleep apnea symptoms: night	3.1 ± 0.7	1.8-4.7	3.2 ± 0.8	1.8-4.7	2.9 ± 0.6	1.9-3.9	.30
Sleep apnea symptoms: day	2.6 ± 0.8	0.9-4.7	2.6 ± 0.9	0.9-4.7	2.7 ± 0.8	1.4-4.6	.75
Epworth Sleepiness Scale	12.6 ± 5.8	4-23	13.7 ± 5.8	4-23	11.7 ± 4.4	2-20	.25
Sleepiness visual analog scale	5.8 ± 2.6	0-10	5.6 ± 3.0	0-10	6.1 ± 2.3	2-10	.77
Functional Outcomes of Sleep Questionnaire	13.8 ± 3.8	6.2-19.3	13 ± 4.5	5.3-19.3	14.2 ± 3.4	3.9-20	.54
Treatment period (number days monitored)	59.6 ± 4.0	43-63	60 ± 3.0	51-63	60.2 ± 2.8	55-63	.26

^*^ *t* test comparing TCC vs UCC

### CPAP Compliance

Participants were followed for an average treatment period of 60 ± 4.0 days. The primary outcome measure of the study was level of CPAP treatment compliance: the TCC group used CPAP 4.1 ± 1.8 hours per night, while the UCC control group used CPAP 2.8 ± 2.2 hours per night (*P* = .07). This difference represented an effect size of 0.65, with membership in the telemonitored group resulting in a 46% increase in CPAP use relative to usual care. There was also a trend for the groups to differ on the percentage of nights with any amount of CPAP use (TCC: 78% ± 22%; UCC: 60% ± 32%; *P* = .07). Table 5 provides details on other measures of CPAP compliance and shows that while none reached statistical significance between study arms, all were in the direction of greater CPAP use in the TCC group.

**Table 5:** CPAP compliance and efficacy data summary, postintervention

	Total Group		TCC		UCC		
Characteristic	Mean ± SD	Range	Mean ± SD	Range	Mean ± SD	Range	*P* value^*^
**Compliance**							
Use (hours/night) (all days)	3.5 ± 2.1	0.2-6.8	4.1 ± 1.8	0.12-6.8	2.8 ± 2.2	0.2-6.2	.07
Use (hours/night) (days used)	4.4 ± 2.2	0.4-8.7	5.0 ± 1.8	0.5-7.7	3.8 ± 2.3	0.4-7.8	.10
% nights with CPAP use > 0 hours of use	65 ± 31	0-100	78 ± 22	24-98	60 ± 32	5-100	.07
% nights with CPAP use > 4 hours of use	44 ± 32	0-93	52 ± 27	0-93	37 ± 34	0-89	.16
**Efficacy**							
Apnea-Hypopnea Index (events/hour)	6.8 ± 5.3	0.1-25.6	7.9 ± 4.1	3.4-19.1	5.0 ± 4.0	0.1-13	.04
Arousal Index (events/hour)	1.5 ± 2.2	0.1-11.6	1.4 ± 1.3	0.2-4.9	1.2 ± 1.4	0.02-4.0	.64
Apnea-Hypopnea Index change	35.6 ± 16.4	12.5-89.3	38.1 ± 18.4	18.6-89.3	32.2 ± 14.8	12.5-58.8	.32
Mask leak, median (L/s)	0.18 ± .36	0-2.1	0.12 ± 0.11	0.01-0.44	0.26 ± 0.51	0-2.1	.29
Mask leak, 95th percentile (L/s)	0.44 ± .36	0.1-2.2	0.38 ± 0.18	0.17-0.82	0.50 ± 0.47	0.1-2.2	.31
Mask leak, maximum (L/s)	0.71 ± .41	0.1-2.2	0.68 ± 0.25	0.26-1.4	0.75 ± 0.54	0.1-2.2	.62

^*^ *t* test comparing TCC vs UCC

### CPAP Efficacy

The two classes of CPAP efficacy measures were effect of CPAP on disease severity (AHI) and effect of CPAP on reduction of mask leak. The mean AHI across the 2-month intervention period was significantly different between the groups (TCC: 7.9 ± 4.1; UCC: 5.0 ± 4.0; *P* = .04), with the TCC group having higher AHI values. However, a more appropriate analysis than direct mean comparison is to take into account the baseline AHI by calculating the reduction in AHI from baseline. Therefore, change in AHI was calculated as baseline AHI (as measured by diagnostic overnight sleep study) minus the mean AHI (as measured by the flow generator device over the course of the intervention period). Table 5 shows the results of the *t* test for change in AHI; there was no statistically significant difference between the groups (TCC: 38.1 ± 18.4; UCC: 32.2 ± 14.8; *P* = .32).

With respect to the effect of CPAP on mask leakage, no statistically significant differences in mask leak level were found between the groups on three difference measures: median leak, 95th percentile leak, and maximum leak. See Table 5 for more details.

### Outcomes

No statistically significant differences were found for any of the outcome measures, as summarized in Table 6. The TCC group had slightly higher scores on the communication with health care team measure than the UCC group (3.3 vs 2.5; *P* = .07).

**Table 6:** Outcome measures, postintervention

	Total Group		TCC		UCC		
Characteristic	Mean ± SD	Range	Mean ± SD	Range	Mean ± SD	Range	*P* value^*^
Sleep apnea symptoms: night	2.4 ± 0.62	1.4-4.0	2.4 ± 0.66	1.4-3.7	2.3 ± 0.61	1.4-4.0	.96
Sleep apnea symptoms: day	2.1 ± 0.89	0.6-4.1	2.0 ± 1.1	0.57-4.1	2.1 ± 0.65	0.92-3.21	.57
Epworth Sleepiness Scale	9.6 ± 5.9	2.0-21.0	9.2 ± 6.6	2.0-21.0	9.9 ± 5.2	3.0-20.0	.72
Sleepiness visual analog scale	4.3 ± 2.9	0.0-10.0	3.8 ± 3.4	0.0-10.0	4.8 ± 2.2	2.0-9.0	.35
Functional Outcomes of Sleep Questionnaire	14.8 ± 4.6	1.8-19.8	15.2 ± 5.0	1.8-19.8	14.4 ± 4.2	1.8-19.9	.61
Center for Epidemiological Studies Depression Scale	8.5 ± 6.4	0.0-23.0	8.6 ± 7.0	0.0-23.0	8.3 ± 5.8	0.0-19.0	.91
CPAP self-efficacy	3.8 ± 1.1	1.0-5.0	4.0 ±1.2	1.0-5.0	3.5 ± 0.83	2.0-5.0	.21
Communication with health care team	2.9 ± 1.2	0.7-5.0	3.3 ± 1.2	0.7-5.0	2.5 ± 1.2	0.7-5.0	.07

^*^ *t* test comparing TCC vs UCC

### Patient Satisfaction

Patients in the TCC group rated their likelihood to continue using CPAP significantly higher than the UCC group (4.8 vs 4.3; *P* = .05). The TCC and UCC groups did not differ in their ratings of satisfaction with care (both groups highly satisfied) or their concerns about being wirelessly monitored (both groups not concerned).

## Discussion

The results of this pilot study suggest that use of telemonitored CPAP compliance and efficacy data to guide the collaborative clinical management of CPAP treatment appears to be as good as usual care in its effect on compliance rates and outcomes in new CPAP users. Given that CPAP use patterns are established early in the treatment initialization process, the monitoring of compliance and efficacy data early in this process, updated in continuous 24-hour periods, to oversee and, if necessary, intervene in treatment is one method clinicians have available to them.

While the usual care compliance rate of 2.8 hours per night is low compared to other published CPAP compliance intervention studies [14-16], it is within close range of compliance levels reported in a comparable studies of the veteran population [34,35], which is on the order of 3.1 hours per night. The TCC group in the present study had a compliance rate of 4.1 hours per night after the 2-month period, which represents a 46% increase in compliance over the mean compliance level of the UCC group (2.8 hours per night) and is equivalent to an effect size of 0.65. According to Cohen’s classification of effect sizes, this would represent a large effect [36].

This study did not find a statistically significant effect at the .05 alpha level for the TCC group on the primary outcome measure of CPAP compliance. The initial power analysis, described above in the Methods section, underestimated the residual variance. With the actual variance, a sample of the size that we collected had a low power, implying that a “failure to reject” at the .05 alpha level results in an unacceptably high type two error rate. The obtained effect size would be clinically significant if it could be established to be reliable, and the only way to do that is to perform a study with a larger sample, as we propose.

The present study confirmed the 100% accuracy rate of wireless data transmission in comparison to manually downloaded data. There was some concern at study outset over the potential for data loss via wireless transmission; however, we found the loss was negligible. While some data may not have come through on the website (either due to residence location with intermittent coverage or to initial patient problems in attaching the units), the data were always available on the flow generator unit itself. It should be noted that once the wireless unit was properly attached, data from previous nights that are stored on the flow generator device can be re-transmitted and obtained wirelessly. As a further safeguard, data can always be manually downloaded directly from the flow generator device during a patient visit. Our experience was that the data could be obtained within a few days and did not negatively impact clinical management. Future generations of flow generator devices may benefit from the pre-installation of an internal wireless device, thereby avoiding potential patient problems with attaching the wireless unit, especially for older adult patients with less technical experience or limited manual dexterity.

The study design dictated that both groups have ResTraxx wireless devices attached to their flow generator units. Prior research has suggested that the mere use of medical devices or components can result in a placebo effect [37]. As such, the UCC group did not necessarily receive usual and customary care because of the presence of the wireless unit. The main implication of this difference is that this study may have underestimated the effect of TCC on CPAP compliance and efficacy measures compared to true usual care. To control and better assess this effect, a third study arm composed of UCC with no wireless device may be added in our future studies of TCC.

Mask leak was not significantly different between the groups. This result is likely owing to the fact that both groups had median mask leak levels that were within normal limits. Likewise, the AHI measured by the CPAP unit across the intervention period was not significantly different between the two groups. This may be a function of measurement (though there is evidence that the AHI measured by the flow generator device used in this study is quite comparable to the AHI measured by polysomnography [31]), sample size, or even limited recording time. It should be noted that limited recording time can play a role because the number of apneas and hypopneas are only measured during the time periods when the CPAP is used. In the present study, CPAP on-time was lower in the UCC group, so their AHI data may be less reliable.

TCC appeared to have no effect on outcomes relative to UCC. Only communication with the health care team was close to being statistically significant at the .05 alpha level. This measure assesses the frequency with which patients use certain communication behaviors, including preparing a list of questions, asking questions about things they want to know and things they don’t understand about their treatment, and discussing any personal problems that may be related to their illness. Focusing on the content and process of patient-sleep provider interactions in future CPAP compliance studies may prove to be a fruitful area of research, particularly in looking at the differences between face-to-face interactions compared to telephone calls.

It can be difficult to reliably measure outcomes in OSA patients given the heterogeneity of the clinical presentation. For example, it is well documented that measures of sleepiness and the current gold-standard measure of disease severity, the AHI, correlate only modestly in the 0.30 range [38]. This is largely due to the fact that OSA is a syndrome, so better characterization of subsets of OSA patients (ie, patient stratification by syndrome composition and severity) would allow more precise outcome measurement. Stratified sampling could not be conducted in the present pilot study owing to the small sample size. We did, however, conduct secondary analyses of AHI groups (moderate: AHI 15-29.99; severe: AHI ≥ 30), but all of the reported findings held within each group.

The present study is most comparable to three prior CPAP telemedicine studies. One study utilized a daily computer-based telephone system to monitor patients’ self-reported compliance behavior and provide automated counseling through a structured dialogue [39]. The impact of the intervention was not significant in comparison to usual care; however, the findings suggest that concurrent education and reinforcement during the initial and early treatment period are effective countermeasures to patient-reported attenuated compliance. Another study utilized computers to provide daily Internet-based informational support and feedback for problems experienced with CPAP use, again without objective measures of compliance, but found no significant differences between the telemedicine intervention and usual care group at 30 days in patient functional status and satisfaction with CPAP [40]. A third study pilot-tested a teleconferencing intervention in which a nurse visually assessed mask fit and patients’ CPAP procedures and provided counseling and reinforcement to a small sample of patients who were trying CPAP again after an initial 3-month period of poor compliance [41]. Although the patient education materials supplied during the initial period did not impact compliance rates, the nurse teleconferencing sessions during the second trial period significantly improved the compliance of the intervention group, suggesting that intensity of one-on-one counseling and feedback by a care provider is a salient variable.

This study was designed as a pilot exploration of the telemedicine intervention in comparison to the standard of care for OSA. Clearly, larger well-powered studies are necessary to follow up on the trends found in the data and to evaluate fully the effect of telemonitoring on compliance, efficacy, and key outcomes in this population.

## Multimedia Appendix

Screenshots of the ResTraxx Data Center website
					

## Abbreviations

AHI: Apnea-Hypopnea Index
CPAP: continuous positive airway pressure
OSA: obstructive sleep apnea
PAP: positive airway pressure
TCC: telemonitored clinical care
UCC: usual clinical care
VASDHS: Veterans Affairs San Diego Healthcare System
