# Supplementary material for: Pilot Randomized Trial of the Effect of Wireless Telemonitoring on Compliance and Treatment Efficacy in Obstructive Sleep Apnea
Source: J Med Internet Res. 2007 May 17;9(2):e14. doi: 10.2196/jmir.9.2.e14 (PMC1874716; doi:10.2196/jmir.9.2.e14)
Supplement: Supplementary file 1 [file jmir_v9i2e14_app1.pdf]

J Med Internet Res
Vol 9 / Iss 2 / e14
Pilot Randomized Trial of the Effect of Wireless Monitoring on Compliance and Treatment Efficacy for Patients With Obstructive Sleep Apnea

Multimedia Appendix

Patient Demographics Tab

This is the first of four tabs that comprise the ResTraxx Data Center. The Patient Demographics tab allows for important demographic data of each patient to be entered, as well as sleep clinic location, health care provider, and identification number. The asterisk indicates required fields, so the end user has the option of how much or how little data to enter in this tab.

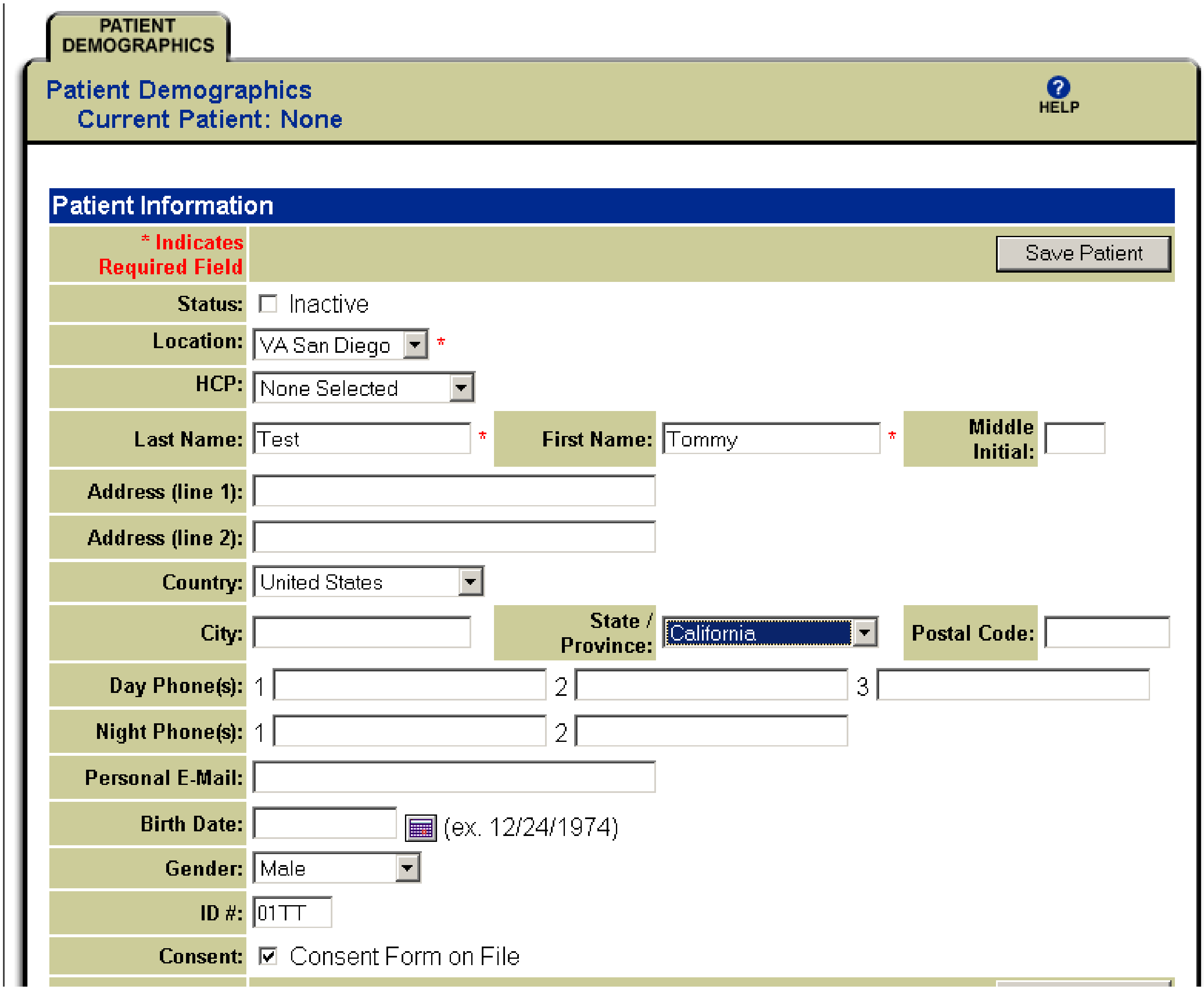

Prescription Tab
This tab allows the sleep clinic to specify the thresholds it would like to monitor for each of several measures, including Apnea-Hypopnea Index, mask leak, and usage. How these threshold levels are used can be seen on the fourth tab, which is called Compliance. Any changes to the threshold level are marked by date in the Prescription History section at the bottom of this tab.

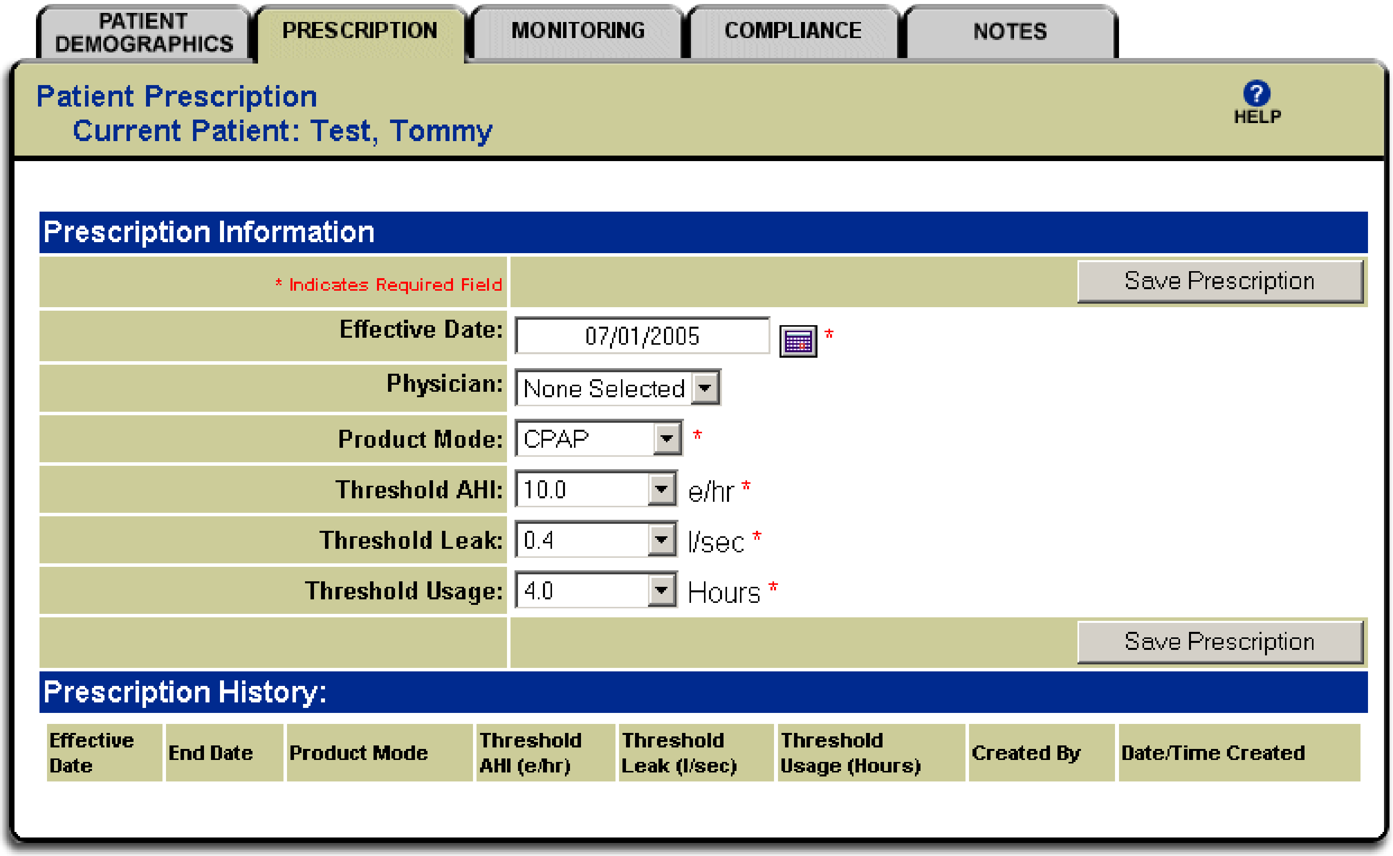

Monitoring Tab

This tab allows for the specification of the start and end of the monitoring period. In addition, this tab allows the sleep clinic to enter both the ResTraxx and flow generator serial numbers, which links the patients with their data. Should data not come through on any one day, the sleep clinic is able to Request History on this tab, which allows the data to be re-sent. The Monitoring History section at the bottom of this tab stores all entered dates for easy review.

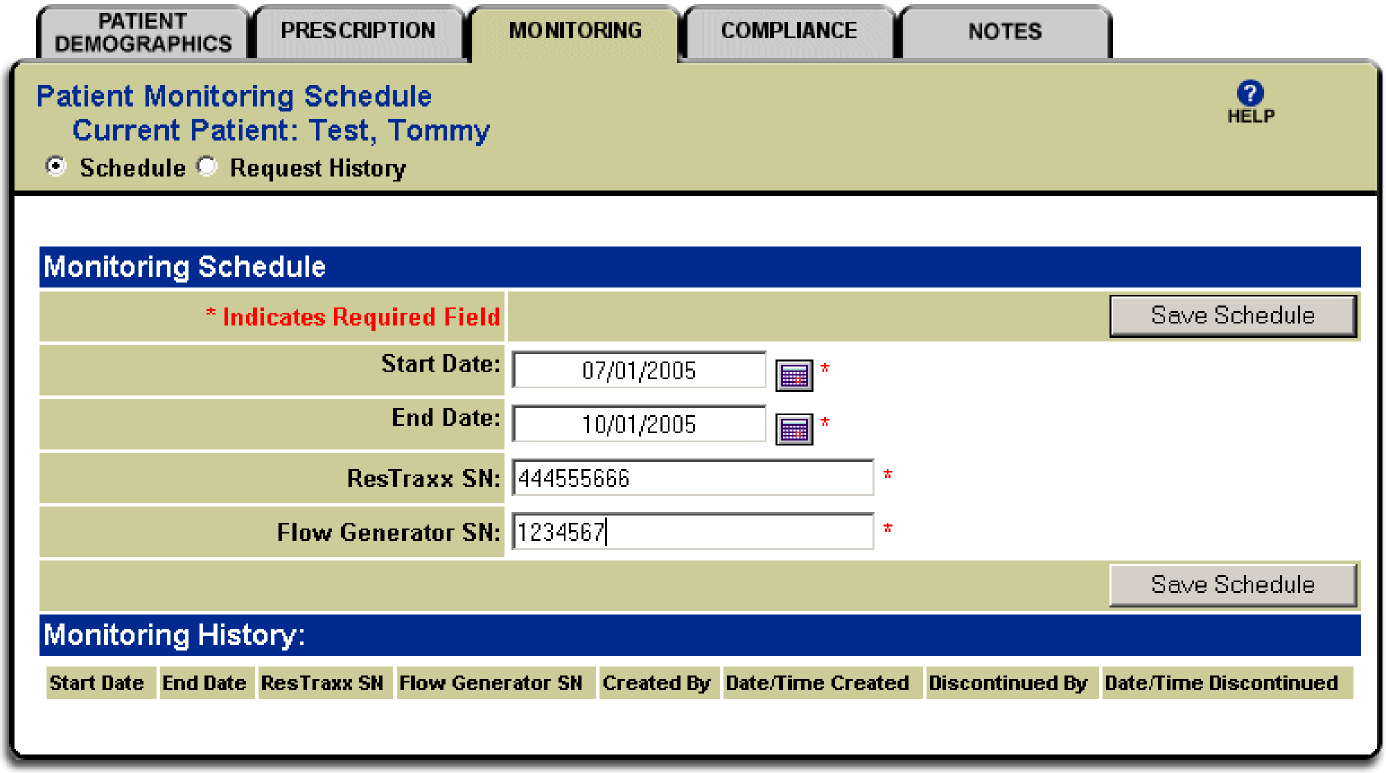

Compliance Tab

This tab allows for the quick visual review of compliance and efficacy data. The screen shot below shows sample compliance and efficacy data for one month of CPAP use for a hypothetical patient. The thresholds specified on an earlier tab allow for color-coding of efficacy and compliance data—this color-coding allows for quick review of how well any one patient is doing on CPAP. Specific data values are also provided, which can aid clinical management. The

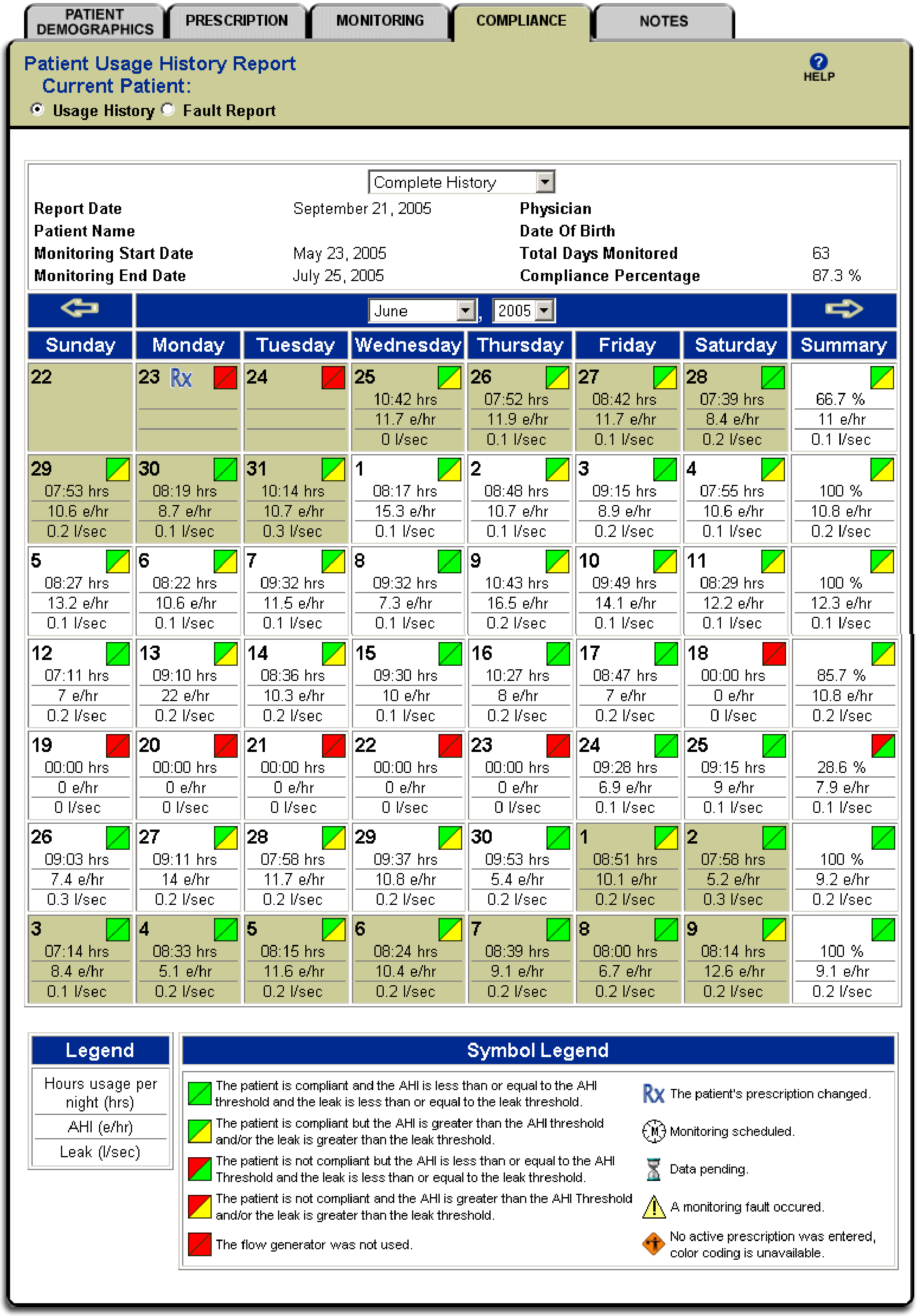

PATIENT DEMOGRAPHICS | PRESCRIPTION | MONITORING | COMPLIANCE | NOTES

Patient Usage History Report

Current Patient:

Usage History / Fault Report

HELP

Complete History

**Report Date**	September 21, 2005	**Physician**	
**Patient Name**		**Date Of Birth**	
**Monitoring Start Date**	May 23, 2005	**Total Days Monitored**	63
**Monitoring End Date**	July 25, 2005	**Compliance Percentage**	87.3 %

June, 2005

Sunday	Monday	Tuesday	Wednesday	Thursday	Friday	Saturday	Summary
22	23 Rx	24	25<br>10:42 hrs<br>11.7 e/hr<br>0 l/sec	26<br>07:52 hrs<br>11.9 e/hr<br>0.1 l/sec	27<br>08:42 hrs<br>11.7 e/hr<br>0.1 l/sec	28<br>07:39 hrs<br>8.4 e/hr<br>0.2 l/sec	66.7 %<br>11 e/hr<br>0.1 l/sec
29<br>07:53 hrs<br>10.6 e/hr<br>0.2 l/sec	30<br>08:19 hrs<br>8.7 e/hr<br>0.1 l/sec	31<br>10:14 hrs<br>10.7 e/hr<br>0.3 l/sec	1<br>08:17 hrs<br>15.3 e/hr<br>0.1 l/sec	2<br>08:48 hrs<br>10.7 e/hr<br>0.1 l/sec	3<br>09:15 hrs<br>8.9 e/hr<br>0.2 l/sec	4<br>07:55 hrs<br>10.6 e/hr<br>0.1 l/sec	100 %<br>10.8 e/hr<br>0.2 l/sec
5<br>08:27 hrs<br>13.2 e/hr<br>0.1 l/sec	6<br>08:22 hrs<br>10.6 e/hr<br>0.1 l/sec	7<br>09:32 hrs<br>11.5 e/hr<br>0.1 l/sec	8<br>09:32 hrs<br>7.3 e/hr<br>0.1 l/sec	9<br>10:43 hrs<br>16.5 e/hr<br>0.2 l/sec	10<br>09:49 hrs<br>14.1 e/hr<br>0.1 l/sec	11<br>08:29 hrs<br>12.2 e/hr<br>0.1 l/sec	100 %<br>12.3 e/hr<br>0.1 l/sec
12<br>07:11 hrs<br>7 e/hr<br>0.2 l/sec	13<br>09:10 hrs<br>22 e/hr<br>0.2 l/sec	14<br>08:36 hrs<br>10.3 e/hr<br>0.2 l/sec	15<br>09:30 hrs<br>10 e/hr<br>0.1 l/sec	16<br>10:27 hrs<br>8 e/hr<br>0.2 l/sec	17<br>08:47 hrs<br>7 e/hr<br>0.2 l/sec	18<br>00:00 hrs<br>0 e/hr<br>0 l/sec	85.7 %<br>10.8 e/hr<br>0.2 l/sec
19<br>00:00 hrs<br>0 e/hr<br>0 l/sec	20<br>00:00 hrs<br>0 e/hr<br>0 l/sec	21<br>00:00 hrs<br>0 e/hr<br>0 l/sec	22<br>00:00 hrs<br>0 e/hr<br>0 l/sec	23<br>00:00 hrs<br>0 e/hr<br>0 l/sec	24<br>09:28 hrs<br>6.9 e/hr<br>0.1 l/sec	25<br>09:15 hrs<br>9 e/hr<br>0.1 l/sec	28.6 %<br>7.9 e/hr<br>0.1 l/sec
26<br>09:03 hrs<br>7.4 e/hr<br>0.3 l/sec	27<br>09:11 hrs<br>14 e/hr<br>0.2 l/sec	28<br>07:58 hrs<br>11.7 e/hr<br>0.2 l/sec	29<br>09:37 hrs<br>10.8 e/hr<br>0.2 l/sec	30<br>09:53 hrs<br>5.4 e/hr<br>0.2 l/sec	1<br>08:51 hrs<br>10.1 e/hr<br>0.2 l/sec	2<br>07:58 hrs<br>5.2 e/hr<br>0.3 l/sec	100 %<br>9.2 e/hr<br>0.2 l/sec
3<br>07:14 hrs<br>8.4 e/hr<br>0.1 l/sec	4<br>08:33 hrs<br>5.1 e/hr<br>0.2 l/sec	5<br>08:15 hrs<br>11.6 e/hr<br>0.2 l/sec	6<br>08:24 hrs<br>10.4 e/hr<br>0.2 l/sec	7<br>08:39 hrs<br>9.1 e/hr<br>0.2 l/sec	8<br>08:00 hrs<br>6.7 e/hr<br>0.2 l/sec	9<br>08:14 hrs<br>12.6 e/hr<br>0.2 l/sec	100 %<br>9.1 e/hr<br>0.2 l/sec

Legend

Hours usage per night (hrs)

AHI (e/hr)

Leak (l/sec)

Symbol Legend

- The patient is compliant and the AHI is less than or equal to the AHI threshold and the leak is less than or equal to the leak threshold.
- The patient is compliant but the AHI is greater than the AHI threshold and/or the leak is greater than the leak threshold.
- The patient is not compliant but the AHI is less than or equal to the AHI Threshold and the leak is less than or equal to the leak threshold.
- The patient is not compliant and the AHI is greater than the AHI Threshold and/or the leak is greater than the leak threshold.
- The flow generator was not used.
- Rx The patient's prescription changed.
- Monitoring scheduled.
- Data pending.
- A monitoring fault occured.
- No active prescription was entered, color coding is unavailable.

Week 1

16	17	18 Rx	19	20	21	[illegible]
		04:28 hrs	00:34 hrs	02:53 hrs	00:00 hrs	[illegible]
		10.5 e/hr	1.7 e/hr	9.3 e/hr	0 e/hr	[illegible]
		0.1 l/sec	0 l/sec	0.1 l/sec	0 l/sec	[illegible]

Week 5

13	14	15	16	17	18	[illegible]
05:46 hrs	04:12 hrs	05:53 hrs	00:00 hrs	07:41 hrs	05:43 hrs	[illegible]
4.3 e/hr	4.7 e/hr	4.2 e/hr	0 e/hr	5 e/hr	5 e/hr	[illegible]
0.3 l/sec	0.3 l/sec	0.3 l/sec	0 l/sec	0.3 l/sec	0.3 l/sec	[illegible]
